# Maternal serum preptin levels in the pathogenesis and diagnosis of Gestational diabetes mellitus

**DOI:** 10.5937/jomb0-36287

**Published:** 2023-03-15

**Authors:** Utku Irem Kıraç, Esra Demır, Hanişe Ozkan, Berrak Sahtıyancı, Hafize Uzun, Iskender Ekıncı, Mitat Buyukkaba, Sinem Durmus, Murat Akarsu, Remise Gelisgen, Omur Tabak

**Affiliations:** 1 Health Sciences University, Sancaktepe Prof. Dr. lhan Varank Training and Research Hospital, Department of Internal Medicine, Istanbul, Turkey; 2 Bagcılar Medipol Mega University, Department of Internal Medicine, Istanbul, Turkey; 3 Health Sciences University, Kanuni Sultan Suleyman Training and Research Hospital, Department of Internal Medicine, Istanbul, Turkey; 4 Bahcelievler Haspital, Department of Internal Medicine, Istanbul, Turkey; 5 Istanbul Atlas University, Faculty of Medicine, Department of Medical Biochemistry, Istanbul, Turkey; 6 Bezmialem Vakif University, Faculty of Medicine, Department of Internal Medicine, Istanbul, Turkey; 7 Istanbul University-Cerrahpasa, Faculty of Medicine, Department of Medical Biochemistry, Istanbul, Turkey

**Keywords:** gestational diabetes mellitus, oral glucose tolerance test, preptin, insulin, gestacijski dijabetes melitus, oralni test tolerancije glukoze, preptin, insulin

## Abstract

**Background:**

Gestational diabetes mellitus (GDM) is a metabolic disorder that occurs during pregnancy that increases both maternal and fetal mortality and morbidity. It was investigated whether there is a change in circulating levels of preptin, a new peptide secreted from pancreatic beta cells, due to GDM in pregnant women. The relationship between serum preptin levels with insulin and other metabolic parameters was also evaluated in these subjects.

**Methods:**

Eighty-five patients diagnosed as GDM and 89 healthy pregnant women with 75 mg oral glucose tolerance test (OGTT) was assessed in terms of serum preptin levels.

**Results:**

The serum preptin levels of the GDM group were significantly higher than those of the control group (p=0.001; p < 0.01). For the cutoff value of preptin measurement of 335.3 ng/L, the sensitivity was 97.65%, specificity was 87.64%, positive predictive value was 88.3% and negative predictive value was 97.5%. The risk of developing the disease is 294.273 times higher in patients with preptin level of 335.3 and above.

**Conclusions:**

We think that the reason for the increase in serum preptin levels in GDM is probably the response to glucose. The current results indicate that preptin plays an important role in elucidating the pathology of GDM. In addition, the search for a practical marker for the diagnosis of GDM suggests that the measurement of preptin level is promising.

## Abstract (sr)

**Uvod:**

Gestacijski dijabetes melitus (GDM) je metabolički poremećaj koji se javlja tokom trudnoće i povećava smrtnost i morbiditet i majke i fetusa. Ispitivano je da li po stoji promena u cirkulišućim nivoima preptina, novog peptida izlučenog iz beta ćelija pankreasa, usled GDM kod trudnica. Odnos između nivoa preptina u serumu sa insulinom i drugih metaboličkih parametara je takođe procenjen kod ovih ispitanika.

**Metode:**

Osamdeset pet pacijenata sa dijagnozom GDM i 89 zdravih trudnica sa 75 mg oralnim testom tolerancije glukoze (OGTT) je procenjeno u smislu nivoa preptina u serumu.

**Rezultati:**

Nivoi preptina u serumu GDM grupe bili su značajno viši od onih u kontrolnoj grupi (p=0,001; p < 0,01). Za graničnu vrednost merenja preptina od 335,3 ng/L, osetljivost je bila 97,65%, specifičnost je bila 87,64%, pozitivna prediktivna vrednost je bila 88,3% i negativna prediktivna vrednost je bila 97,5%. Rizik od razvoja bolesti je 294.273 puta veći kod pacijenata sa nivoom preptina od 335,3 i više.

**Zaključak:**

Smatramo da je razlog za povećanje nivoa serumskog preptina u GDM verovatno odgovor na glukozu. Sadašnji rezultati pokazuju da preptin igra važnu ulogu u rasvetljavanju patologije GDM. Pored toga, potraga za praktičnim markerom za dijagnozu GDM-a sugeriše da je merenje nivoa preptina obećavajuće.

## Introduction

Gestational diabetes mellitus (GDM) is glucose intolerance of varying degrees that begins during pregnancy. Although the incidence of GDM in the population varies from 2% to 38%, the prevalence is about 17% worldwide [1]. It is associated with an increased risk of short- and long-term complications for both the baby and the mother during and after pregnancy [2]. Risk factors for GDM include: impaired glucose tolerance in history, GDM in previous pregnancy, family history of diabetes, high BMI, advanced maternal age, and birth of a macrosomic baby in history [3] [4].

There is no complete international consensus on the approach that should be used in the diagnosis and management of GDM. There continues to be debate about whether screening should be done in general or high-risk groups, the timing of screening, the test to be used, and the thresholds for assessment. For screening and diagnosis of gestational diabetes, obstetricians in the United States tend to use the two-step approach regardless of gestational age [5], whereas other countries, including Turkey, generally tend to use a one-step approach [75 mg oral glucose tolerance test (OGGT)] [6]. Because multiple samples must be collected when using the OGTT, there are problems with its use if samples are not given in a timely manner or are left before the process is completed. In addition, nausea and vomiting are commonly observed limitations in the use of the OGGT. Studies highlight the need to develop new and simpler diagnostic methods [7].

A relation was reported between GDM and the circulating levels of some peptide hormones as ghrelin, obestatin, visfatin, preptin. Preptin is a peptide composed of 34 amino acids that is secreted by pancreatic beta cells along with insulin and amylin [8]. Experimental studies have also shown the insulin-releasing effect of preptin. This peptide, which is closely related to insulin resistance, may be important in explaining the pathophysiology of GDM. In the literature, DM has been associated with metabolic syndrome, obesity, polycystic ovary syndrome, hypertension, thyroid disease, and cardiovascular disease [9] [10] [11] [12]. In a small number of studies with limited patient populations, maternal serum and cord blood preptin levels have also been examined and found to be significantly associated with GDM [13].

In our study, it was investigated the relationship between serum preptin level and GDM. In this way, we investigated the role of preptin in the pathophysiology of GDM and whether it could be used as a marker in the diagnosis of GDM in the future.

## Materials and methods

All pregnant subjects who participated in the study gave informed consent and the study was approved by the ethics committee of our hospital (approval number: KAEK/2021.06.213). This study was conducted in accordance with the Declaration of Helsinki.

Our study was conducted on 174 female subjects who presented to Kanuni Sultan Suleyman Training and Research Hospital between August and November 2021. A total of 174 participants, 85 patients and 89 volunteers, were enrolled in the study. The study participants were divided into two groups to assess relevant variables: the GDM patient group and the control group. Pregnant women aged 24-28 weeks who presented to the Internal Medicine Outpatient Department of Kanuni Sultan Suleyman Training and Research Hospital and were diagnosed with GDM by routine OGGT test according to the American Diabetes Association criteria (ADA) were selected as the GDM patient group [6]. The control group consisted of healthy pregnant women aged 24 to 28 weeks who presented to the outpatient clinic for routine examination and were not suffering from any disease. Patients were included in the study if they were over 18 years of age, were between 24 and 28 weeks pregnant, had no previous diagnosis of diabetes, knew of no chronic diseases, and were not taking any medications. Patients with known overt diabetes, hypertension, preeclampsia, active infection, malignant diseases, chronic renal failure (creatinine > 1.5), severe malnutrition, advanced-stage heart failure (stage 3±4), chronic inflammatory diseases, chronic lung diseases (such as COPD, bronchiectasis, asthma, pulmonary hypertension) and thyroid dysfunction were excluded from the study.

The diagnosis of GDM was made by a 75 g OGTT performed between the 24th and 28th week of gestation and in accordance with the American Diabetes Association guidelines (ADA) [6].

Fasting blood glucose was measured after 8 hours of fasting followed by a 75 g OGGT test. GDM was diagnosed based on the assessment of the following fasting blood glucose levels: 5.11 mmol/L, first-hour plasma glucose: 9.99 mmol/L, second-hour plasma glucose: 8.49 mmol/L.

Age, gender, smoking status, height, weight, BMI, waist circumference, hip circumference, medical disease history and medication of the subjects were recorded. Among the comorbid diseases, diabetes mellitus type 2 (T2DM), pre-diabetes, hyper tension, hyperlipidemia and hypothyroidism were specifically queried and recorded. Medications taken by the patients with comorbid diseases were also recorded. Blood pressure was measured in all participants. For determination of preptin level, blood was drawn from the participants and serum samples were stored at -80°C after centrifugation at 3000 rpm (revolutions per minute). Plasma glucose, lipid profiles, aspartate aminotransferase (AST), alanine aminotransferase (ALT), gamma-glutamyl transferase (GGT), and uric acid levels were measured spectrophotometrically using the Abbot Aeroset 2.0 (Abbott Diagnostic, Abbott Park, IL USA). HbA1c (%) was measured using the Variant 2 Turbo (Biorad, Hercules, CA, USA), which uses glycation-specific binding of boron affinity to detect all glycated Hb species present. Complete blood count (CBC) was measured with the Sysmex XN 9000 (Sysmex Europe GmbH, Norderstedt, Germany) hematology analyzer, and insulin levels were measured with the Cobas 8000 C702 (Roche Diagnostics, Indianapolis, IN, USA) chemistry analyzer. Electrolytes were measured using the Cobas 8000 C702 (Roche Diagnostics) chemistry analyzer (Biomolecules 2019, 9, 24 3 of 8). The homeostatic model score for insulin resistance (HOMA-IR) was calculated using the following formula: HOMA-IR = fasting insulin (mIU/mL) x fasting glucose (mmol/L)/22.5.

Serum preptin levels were measured using the Human Preptin ELISA Kit (catalog number: E1448Hu, BT-LAB). The coefficients of intra and inter assay variation were 4.6% (n = 20) and 5.7% (n = 20), respectively.

### Statistical analysis

Using the »comparison of two ratios« formula to determine the sample size and assuming an alpha error of 5%, it was concluded that at least 172 patients should be included to achieve a study power of 80%. A total of 174 participants, 85 patients and 89 volunteers, were included in the study. The Number Cruncher Statistical System (NCSS) 2007 program (Kaysville, Utah, USA) was used for statistical analysis. Descriptive statistical methods (mean, standard deviation, median, frequency, percentage, minimum, maximum) were used to analyze the study data.

Agreement of quantitative data with normal distribution was tested using Shapiro-Wilk test and graphical tests. Independent groups t-test compared normally distributed quantitative variables between two groups and Mann-Whitney U test compared non-normally distributed quantitative variables between two groups. Pearson’s chi-square test and Fisher’s exact test were used to comparing qualitative data. Spearman correlation analysis evaluated the relationships between quantitative variables. Diagnostic screening tests (sensitivity, specificity, PPV, NPV) and ROC analysis were performed to determine the cut-off value for the parameters. Statistical significance was accepted as p < 0.05.

## Results

Age and number of pregnancies of participants in the GDM patient group were not statistically significantly different between groups (p > 0.05). The incidence of GDM in the history was statistically significantly higher among patients in the GDM patient group than in the control group (p=0.035). The BMI values of the participants in the GDM patient group were statistically significantly higher than those of the control group (p=0.027) (Table 1).

**Table 1:** Demographic characteristics of the gestational diabetes mellitus (GDM) and healthy groups. ^a^Student t-Test;^ b^Mann-Whitney U Test; ^c^Pearson Chi-Square Test; ^*^p<0.05;^**^p<0.01

		GDM group<br>(n=85)	Healthy group<br>(n=89)	P
Age<br>(Year)	Mean<br>±SD	30.2±4.9	31.3±6.4	0.169^a^
BMI (kg/m^2^)	Mean<br>±SD	29.37±4.64	27.85±4.33	0.027^a*^
Number of pregnancies	Mean<br>±SD	2.66±1.38	2.4±1.25	0.229^b^
History of GDM	yes	75 (88.2)	86 (96.6)	0.035^c*^
	no	10 (11.8)	3 (3.4)	

The presence of HT, smoking and family history distribution showed no statistically significant difference between groups (p > 0.05) (Table 2).

**Table 2:** Evaluation of chronic disease and smoking status according to groups. ^c^Pearson Chi-Square Test ^d^Fisher’s Exact Test

		GDM group<br>(n=85)	Healthy<br>group<br>(n=89)	P
		n (%)	n (%)	
Hypertension	no	83 (97.6)	88 (98.9)	0.614^d^
	yes	2 (2.4)	1 (1.1)	
Smoking	no	71 (83.5)	75 (84.3)	1.000^c^
	yes	14 (16.5)	14 (15.7)	
Family History of GDM	no	55 (64.7)	46 (51.7)	0.082^c^
	yes	30 (35.3)	43 (48.3)	

The urea, creatinine, AST, LDL, HDL, TG, total cholesterol, uric acid, GGT, HG, WBC, PLT, MPV measurements of the patients showed no statistically significant difference between the groups (p > 0.05). ALT values of the GDM patient group were found to be statistically significantly higher than those of the control group (p=0.009; p < 0.01) (Table 3).

**Table 3:** The laboratory levels of the gestational diabetes mellitus (GDM) and control groups. ^a^Student t-Test; ^b^Mann-Whitney U Test; ^*^p<0,05; ^**^p<0,01, AST – aspratate aminotransferase; ALT – alanine aminotransferase; LDL – low density lipoprotein; HDL – high density lipoprotein; TG – triglyceride; GGT – gamma-glutamyl transferase; Hb – hemoglobin; WBC – white blood counts; MPV – mean platelets volume; HbA_1C_ – hemoglobin A1_C_; HOMA-IR – homeostatic model assessment-insulin resistance; SD. – standard deviation.

		GDM group<br>(n=85)	Healthy group<br>(n=89)	p
Urea (mmol/L)	Ort±SD	5.22±1.42	5.00±1.33	0.283 a
Creatinine (mmol/L)	Mean ±SD	39.78±7.96	38.01±7.10	0.213^b^
Insulin (mU/L)	Ort±SD	11.89±4.59	12.33±5.24	0.832^b^
AST (U/L)	Mean ±SD	17.91±5.95	16.73±4.94	0.161^b^
ALT (U/L)	Mean ±SD	14.33±6.13	12.55±5.93	0.009^b**^
LDL (mmol/L)	Mean ±SD	2.95±1.30	2.90±0.99	0.699^b^
HDL (mmol/L)	Mean ±SD	1.71±0.43	1.75±0.39	0.478^a^
TG (mmol/L)	Mean ±SD	2.33±0.80	2.22±0.85	0.399^a^
T. Cholesterol (mmol/L)	Mean ±SD	5.67±1.55	5.66±1.22	0.951^a^
Uric acid (μmol/L)	Mean ±SD	195±49	194±35	0.929^a^
GGT (IU/L)	Mean ±SD	90.00±54.50	83.70±49.30	0.436^b^
Hgb (g/L)	Mean ±SD	112.10±9.60	110.90±13.30	0.737^b^
WBC (x10^3^)	Mean ±SD	10.25±2.18	9.65±2.28	0.079^a^
PLT (x10^9^)	Mean ±SD	237.71±62.2	241.31±65.33	0.710^a^
MPV (fL)	Mean ±SD	0.9±0.91	10.83±1.27	0.672^a^
Potasium (mmol/L)	Mean ±SD	4.13±0.3	4.04±0.27^a^	0.046^*^
Sodium (mmol/L)	Mean ±SD	137.24±1.74	37.46±1.36	0.684^b^

It was determined that the serum preptin levels of participants in the GDM patient group were statistically significantly higher than those of the control group (p=0.001; p < 0.01) (Table 4).

**Table 4:** The OGTT levels of the gestational diabetes mellitus (GDM) and control groups. OGTT–oral glucose tolerans test; SD.—standard deviation. ^a^Student t-Test; ^b^Mann-Whitney U Test; ^*^p<0.05; ^**^p<0.01

		GDM group<br>(n=85)	Healthy group<br>(n=89)	p
Glucose (mmol/L)	Mean ±SD<br>Median (Min-Maks)	4.71±0.80<br>4.66 (3.05–8.55)	4.05±0.57<br>4.05 (2.72–5.72)	0.001 a**
OGTT 0. (mmol/L)	Mean ±SD<br>Medyan (Min-Maks)	5.22±0.64<br>5.11 (3.05–8.44)	4.54±0.30<br>4.61 (3.89–5.05)	0.001^b**^
OGTT 1. (mmol/L)	Ort±SD<br>Medyan (Min-Maks)	9.45±2.07<br>9.71 (4.44–14.38)	7.24±1.30<br>7.27 (4.55–9.94)	0.001^a**^
OGTT 2. (mmol/L)	Mean ±SD<br>Medyan (Min-Maks)	8.03±1.70<br>7.99 (1.70–13.26)	5.03±1.04<br>5.77 (3.39–8.21)	0.001^a**^

Based on this significance, it was considered to calculate the cut-off point for preptin. ROC analysis and diagnostic screening tests were used to determine the cut-off point for each group. The cut-off point for preptin measurements was set at 335.3 and above for the study and control groups. For the cut-off value of preptin measurement of 335.3, the sensitivity was 97.65%, specificity was 87.64%, positive predictive value was 88.3% and negative predictive value was 97.5% (Table 4).

**Figure 1:**
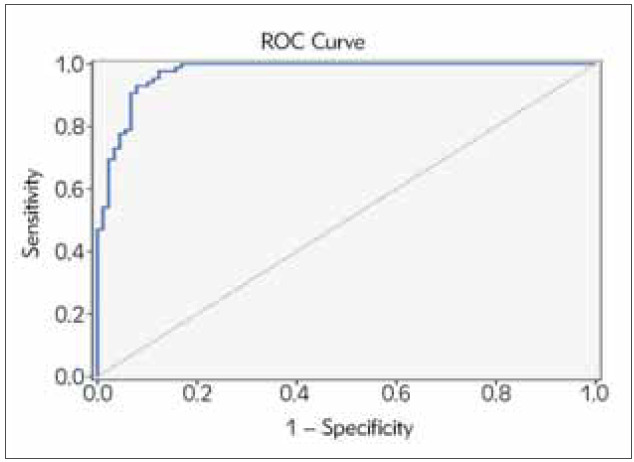
The distribution of thyroid stimulating hormone (TSH) with age. Plots showing the median TSH for each of the changes according to different age group.

The area under the ROC curve obtained was 92.6% with a standard error of 2.3% (Figure 1). The Odds Ratio (OR) for preptin was 294.273 (95% CI: 63.212–1369.937) (Table 5). A statistically significant correlation was found between the groups and the cut-off value of the preptin level of 335.3 (p=0.001). We can say that the risk of developing the disease is 294.273 times higher in patients with preptin level of 335.3 and above. Table 6

**Table 5:** Serum preptin levels between groups. ^b^Mann-Whitney U Test; ^**^p<0.01

		GDM group<br>(n=85)	Healthy group<br>(n=89)	P
Preptin<br>(ng/L)	Mean ±SD	846.28±476.26	256.16±111.15	0.001^b**^
	Medyan<br>(Min-Maks)	736.7<br>(304.2–2378.6)	234.7<br>(158.9–788.4)	

**Table 6:** Diagnostic Screening Tests and ROC Curve Results for serum preptin

	Diagnostic Scan					ROC Curve		P
	Cutoff	Sensitivite	Spesifisite	Positive<br>Predictive Value	Negative<br>Predictive Value	Area	95%<br>Confidence<br>Interval	
Preptin (ng/L)	335.3	97.65	87.64	88.30	97.50	0.926	0.882-0.971	0.001^**^

## Discussion

Although GDM is a condition that resolves immediately with termination of pregnancy, there is no clear data on how it develops during pregnancy. Peptides are thought to play a role in pathogenesis. Preptin is a 34 amino acid peptide that is secreted by pancreatic beta cells along with insulin and amylin.

In a study by Buchanan et al. [14] in a rat pancreas model, preptin was shown to play a direct role in glucose-mediated insulin secretion. In the same study, it was found that glucose-dependent insulin secretion increased by 30% after preptin infusion into rat pancreas. Yang et al. [9] was reported that preptin may play a role in the development of type 2 DM, and it was indicated that preptin levels were significantly higher in the DM group. In current study, we considered the similarities in the pathogenesis of type 2 DM and GDM and hypothesized that preptin might also play a role in the development of GDM. There are few studies in the literature investigating preptin levels in serum, cord blood and colostrum of GDM patients. In a study conducted by Aydin et al. [15] in 36 patients, 12 of whom had GDM, it was found that preptin levels in plasma and colostrum were significantly higher in the GDM group. In another study conducted by Aslan et al. [13] in 62 patients, 31 of whom had been diagnosed with GDM, it was revealed that preptin levels in maternal plasma and fetal cord blood were significantly higher in the GDM group. On the other hand, in a study by Ersahin et al. [16] difference was not found between the preptin levels in plasma of GDM patients and healthy pregnant women with the same BMI [16].

In current study, serum preptin level was significantly higher in the GDM patient group than in the control group. This increase may be due to either increased secretion of preptin or decreased metabolism. Again, we detected that the cut-off point for preptin measurements in the GDM patient and control groups was 335.3 and above. We can say that the risk of disease is 294.273 times higher at preptin level of 335.3 and above. This result gave us the idea that measuring preptin level could be a practical indicator for diagnosing GDM in the future. We did not find similar data in the literature. The OGGT test is routinely performed for the diagnosis of GDM. However, there are problems such as not being able to finish the drink completely, not being able to continue the test, not giving the blood sample at the right time, and ending the test due to nausea and vomiting [17].

It may be beneficial to have a marker in the blood that can be tested immediately once. In this regard, it is recommended that testing be performed in larger groups of patients. Furthermore, no correlation was found between preptin levels and BMI in our study’s GDM patient and control groups. This suggested that a significant increase in preptin level in the GDM population was not associated with increased BMI. Baykus et al. [18] found that A positive correlation was established between desacylated ghrelin and acylated ghrelin, desacylated ghrelin and preptin and preptin and insulin in the GDM group during pregnancy. Preptin is reported to be secreted together with insulin in response to glucose [19].

We think that the reason for the increase in serum preptin levels in GDM is probably the response to glucose. In future studies, measuring serial changes in serum preptin levels from the beginning of pregnancy may provide valuable clues to elucidate the role of preptin in the pathogenesis of GDM. Further studies are needed to investigate the role of this peptide in the pathogenesis of GDM. It could be used in the diagnosis of GDM in the future.

## Dodatak

### Acknowledgments

This research did not receive any specific grant from any funding agency in the public commercial or a non-profit section.

### Conflict of interest statement

All the authors declare that they have no conflict of interest in this work.
